# How does cellulosome composition influence deconstruction of lignocellulosic substrates in *Clostridium* (*Ruminiclostridium*) *thermocellum* DSM 1313?

**DOI:** 10.1186/s13068-017-0909-7

**Published:** 2017-09-18

**Authors:** Shahar Yoav, Yoav Barak, Melina Shamshoum, Ilya Borovok, Raphael Lamed, Bareket Dassa, Yitzhak Hadar, Ely Morag, Edward A. Bayer

**Affiliations:** 1 Department of Plant Pathology and Microbiology, Robert H. Smith Faculty of Agriculture, Food and Environment, The Advanced School for Environmental Studies, The Hebrew University of Jerusalem, 76100 Rehovot, Israel; 2 Designer Energy Ltd, 2 Bergman Street, Rehovot, Israel; 3 Bio-Nano Unit, Chemical Research Support, The Weizmann Institute of Science, 761000 Rehovot, Israel; 4 Department of Biomolecular Sciences, The Weizmann Institute of Science, 76100 Rehovot, Israel; 5 Department of Molecular Microbiology and Biotechnology, Tel Aviv University, Ramat Aviv, Israel

**Keywords:** Lignocellulosic biomass, Proteomics, Enzymatic hydrolysis, Scaffoldins, Biofuels

## Abstract

**Background:**

Bioethanol production processes involve enzymatic hydrolysis of pretreated lignocellulosic biomass into fermentable sugars. Due to the relatively high cost of enzyme production, the development of potent and cost-effective cellulolytic cocktails is critical for increasing the cost-effectiveness of bioethanol production. In this context, the multi-protein cellulolytic complex of *Clostridium* (*Ruminiclostridium*) *thermocellum,* the cellulosome, was studied here. *C. thermocellum* is known to assemble cellulosomes of various subunit (enzyme) compositions, in response to the available carbon source. In the current study, different carbon sources were used, and their influence on both cellulosomal composition and the resultant activity was investigated.

**Results:**

Glucose, cellobiose, microcrystalline cellulose, alkaline-pretreated switchgrass, alkaline-pretreated corn stover, and dilute acid-pretreated corn stover were used as sole carbon sources in the growth media of *C. thermocellum* strain DSM 1313. The purified cellulosomes were compared for their activity on selected cellulosic substrates. Interestingly, cellulosomes derived from cells grown on lignocellulosic biomass showed no advantage in hydrolyzing the original carbon source used for their production. Instead, microcrystalline cellulose- and glucose-derived cellulosomes were equal or superior in their capacity to deconstruct lignocellulosic biomass. Mass spectrometry analysis revealed differential composition of catalytic and structural subunits (scaffoldins) in the different cellulosome samples. The most abundant catalytic subunits in all cellulosome types include Cel48S, Cel9K, Cel9Q, Cel9R, and Cel5G. Microcrystalline cellulose- and glucose-derived cellulosome samples showed higher endoglucanase-to-exoglucanase ratios and higher catalytic subunit-per-scaffoldin ratios compared to lignocellulose-derived cellulosome types.

**Conclusion:**

The results reported here highlight the finding that cellulosomes derived from cells grown on glucose and microcrystalline cellulose are more efficient in their action on cellulosic substrates than other cellulosome preparations. These results should be considered in the future development of *C. thermocellum*-based cellulolytic cocktails, designer cellulosomes, or engineering of improved strains for deconstruction of lignocellulosic biomass.

**Electronic supplementary material:**

The online version of this article (doi:10.1186/s13068-017-0909-7) contains supplementary material, which is available to authorized users.

## Background

Developing cost-effective and renewable alternative energy resources capable of replacing currently used fossil fuel is an important challenge [1]. Cellulosic ethanol, one of the suggested solutions of this global issue, meets the necessary requirements of being renewable and environmentally friendly [2, 3]. Unlike the production process of the first-generation bioethanol alternative, which utilizes the edible parts of plants, the cellulosic ethanol alternative exploits the inedible polysaccharides of the plant, notably the cellulose, found in the cell walls of lignocellulosic biomasses [4, 5]. Agriculture or industrial lignocellulosic wastes can be used as sources of biomass, although removal of plant residues from the field could also have negative effects on soil fertility and quality [6].

The plant cell wall is a chemically complex structure composed of cellulose, hemicelluloses, and lignin as the main polymers. Those polymers, together with other components, provide the plant cell with the robustness required for its diverse functions [7, 8].

The production process for converting cellulosic biomass to ethanol involves three major steps [2]. The first includes chemical or physical pre-treatment, which is designed to loosen the rigid structure of the plant cell wall, to increase cellulose accessibility and to enrich the cellulose fraction. In the second step, the enriched cellulose fraction is hydrolyzed into soluble fermentable sugars. In the third step, the soluble sugar mixture is used as a carbon source for alcoholic fermentation. To date, the hydrolysis step is performed by enzymatic hydrolysis, rendering cellulosic ethanol economically infeasible, mainly due to the relatively high production costs of the carbohydrate-hydrolyzing enzymes [9, 10].

Cellulolytic microorganisms can utilize the cellulose as a carbon source. The natural arsenal of plant cell wall-degrading enzymes is diverse, and includes cellulases, hemicellulases, pectinases, ligninases, and additional accessory enzymes. Cellulose-hydrolyzing enzymes are classified into three major groups by their sequence homology and biochemical characteristics: (A) endoglucanases, which cleave bonds in the middle of the cellulose chains in random or semi-random fashion; (B) exoglucanases, which hydrolyze cellulose from either the reducing or non-reducing end in a processive manner, releasing soluble non-monomeric sugars; and (C) β-glucosidases, which hydrolyze the end-product (cellobiose) of cellulase hydrolysis to produce glucose [11, 12].

*Clostridium thermocellum* (recently reclassified as *Ruminiclostridium thermocellum*) is one of the best-explored and well-characterized cellulose-degrading bacteria in nature. Due to its characteristics, this anaerobic thermopilic bacterium was suggested to be the organism of choice for bioethanol production processes [13–17]. Its cellulolytic machinery, called cellulosome, is a multi-protein complex that contains a multiplicity of catalytic subunits, as well as structural proteins (scaffoldins) which are responsible for integrating the catalytic subunits into a well-ordered high-molecular-weight complex [14, 18]. Selected scaffoldins can bind cellulose by virtue of an integral cellulose-binding module (CBM), which are attached to the bacterium via an anchoring protein that contains an S-layer homology (SLH) module [19]. In this manner, the cellulosome creates proximity and substrate-targeting effects [20]. There are more than eighty genes in the genome of *C. thermocellum* that encode for cellulosomal subunits [21]. In addition to the cellulosome, *C. thermocellum* also utilizes soluble non-cellulosomal cellulolytic enzymes for deconstruction of cellulose [22–24].

The composition and structure of plant cell walls differ among different plant species as well as among different tissues in a given plant. In addition to their inherent variability, different pre-treatments can further alter the composition of the lignocellulosic biomass, leading to even higher diversity among the carbon sources used in the bioethanol production process [8]. Consequently, different enzyme compositions might be required for efficient hydrolysis of the different carbon sources. Indeed, proteomic and transcriptomic studies have shown that the expression pattern of the cell wall-degrading enzymes and the composition of the cellulosomes change in response to the carbon source of the medium. In other words, *C. thermocellum* senses the biomass in the medium and assembles a cellulosome preparation tailored to the requirements of the bacterium. Former studies have highlighted the need for understanding differential assembly of cellulosomal subunits in order to reveal key enzymes that are important for efficient hydrolysis [25–28].

In the current study, cellobiose (CB), microcrystalline cellulose (MCC), alkaline-pretreated switch grass (alSG), alkaline-pretreated corn stover (alCS), and dilute acid-pretreated corn stover (acCS), were used as sole carbon sources for growth of *C. thermocellum* DSM1313. In nature, *C. thermocellum* hydrolyzes cellulose into cellobiose units, which are in turn consumed by the bacterium. An adaptation process can enable some *C. thermocellum* strains to utilize monomeric glucose as a sole carbon source [13, 29–31]. In this study, such an adaptation process was conducted, and glucose was also used as a sole carbon source. The influence of various carbon sources on the structure and subunit composition of the resultant cellulosomes, and consequently on its hydrolysis activity, was investigated.

## Methods

### Alkaline pre-treatment of corn stover and switchgrass

Corn stover was collected after harvest from Moshav Kfar HaRif, Israel. Switchgrass was obtained from Notts Farms, Clinton, ON, Canada. Alkaline pre-treatment was carried out as previously described [32]. Briefly, 100 g of each feedstock were separately placed into 2-L glass beakers followed by the addition of 700 mL of 2% [wt/wt] NaOH solution. The beakers containing biomass and alkali solution were heated to boiling and allowed to proceed under this temperature for 1 h with continuous stirring. The pretreated biomasses were then washed by water through a glass Buchner funnel and adjusted to neutral pH. Finally, the pretreated biomasses were drained using vacuum through the funnel and brought to about 20–30% (wt/wt) solid content.

### Dilute acid-pretreated corn stover

Dilute sulfuric acid-pretreated corn stover **(**160 °C for 1 min at an effective acid concentration of 1–2% [w/w]) was obtained from the National Renewable Energy Laboratory (NREL), Golden, CO (Batch Number P080828-CS-8.‬ Manufactured: 26.11.13; Record No. 579)‬‬‬‬‬‬‬‬‬‬‬‬‬‬‬‬‬‬.

### Chemical composition of biomass

The chemical composition of the various biomasses was determined by conventional chemical analysis methods [33, 34]. Briefly, for delignification, the desired lignocellulosic biomass (1% slurry [w/v]) was supplemented with 1% acetic acid [v/v] and 1.5% [w/v] sodium chlorite and boiled for 1 h. The delignification process was then repeated. The obtained (white) holocellulose, i.e., complex of cellulose and hemicelluloses, was hydrolyzed by boiling in 1.5% hydrochloric acid for 2 h. The content of cellulose was calculated from the dry residue remaining after hydrolysis of the holocellulose, while the content of hemicelluloses was measured from weight loss of the hydrolyzed holocellulose sample. Lignin Klason was analyzed by means of standard TAPPI procedure T222 [34].

### Anaerobic fermentation of *C. thermocellum*

Growth of the anaerobic thermophilic bacterium *C. thermocellum* (strain DSM 1313 obtained from the DSMZ collection) was performed as previously described [35] with minor changes. Briefly, GS-2 medium (0.5 g/L K_2_HPO_4_, 0.5 g/L MgCl_2_·6H_2_O, 0.5 g/L KH_2_PO_4_, 1.3 g/L (NH4)_2_SO_4_, 0.002 g/L resazurin, 10.5 g/l 3-(*N*-morpholino) propanesulfonic acid (MOPS) buffer, 5 g/L yeast extract, 0.5 mM CaCl_2_, and 1.25 mg/L iron(II) sulfate) was adjusted with 10 M NaOH to a final pH of 7.2. A portion (400 mL) of the medium was transferred into 0.5 L serum bottles containing 0.3% (wt/vol) of the different carbon sources (except 0.5% in the case of glucose), boiled, and extensively flushed with nitrogen. The bottles were sealed, autoclaved (121 °C, 20 min), and inoculated with a fresh CB-based *C. thermocellum* starter culture. For inoculation of glucose-containing media, a preliminary adaptation process was performed. Glucose-based media were thus inoculated with *C. thermocellum* followed by seven successive re-inoculation steps, which resulted in significant shortening of the lag phase rendering it comparable to the that of cellobiose-based growth media. Triplicate samples were prepared. Bottles were incubated for 48 h in a 60 °C shaking incubator.

### Cellulosome purification

*C. thermocellum* growth media were centrifuged (10,900*g*, 7 min), and the supernatant fluids were carefully removed from the pellet and concentrated 40 times using a Pellicon XL biomax 300 cassette (Millipore, Cat. No. PXB300C50). Concentrated samples were fractionated by size exclusion chromatography using a Superdex S-200 prep grade 16/60 gel filtration column (GE Healthcare). Fractions (1 mL) were collected and analyzed by 6% SDS-PAGE. Fractions containing the cellulosomes (scaffoldin and identified enzymatic subunits) were pooled (Additional file 1: Figure S1). Cellulosome concentration was determined using a Pierce™ BCA Protein Assay Kit (Thermo scientific, Waltham, MA). Since samples from cellobiose- and glucose-based media showed relatively low concentrations, they were further concentrated by Vivaspin (Sartorius, Goettingen, Germany) with polyethylene sulfate (PES) membrane (30,000 MWCO). Samples were stored at −20 °C until use.

### β-glucosidase

*Thermoanaerobacter brockii* thermostable β-glucosidase, CglT (GenBank: ADV80605.1), was a kind gift of CelDezyner LTD, Israel (alon@celdezyner.com). The concentration of CglT in the unpurified sample was determined based on comparative activity tests (1 mL of 50 mM sodium citrate buffer, pH 6.0, containing 5 mM *p*-nitrophenyl-β-d-1, 4-glucopyranoside [Sigma-Aldrich, Rehovot, Israel] was supplemented with 5 µL CglT sample dilutions, followed by incubation at 60 °C for 10 min. Optical densities were measured at a wavelength of 405 nm and compared to that of an assay mixture containing purified CglT.

### Activity assay

All activities assays were conducted in a final volume of 1 mL solution, containing 20 mM citrate buffer (pH 6.0) supplemented with 10 mM CaCl_2_, and substrate loadings of 7% for the MCC hydrolysis assay or 5% for the lignocellulosic biomasses. Cellulosome loadings of 20, 50, 3, or 50 µg/mL were used for MCC, alSG, alCS, and acCS hydrolysis assays, respectively. The latter concentrations were found to be in the near-linear range of the reactions, as determined by preliminary calibration experiments (Additional file 2: Figure S2). CglT (equivalent to 0.33 mg/mL of purified enzyme) was added to the reaction mixture in order to prevent cellobiose feedback inhibition. To evaluate the activities, samples were incubated overnight at 70 °C with continuous shaking, centrifuged, and the supernatant fluids were separated from the undigested biomass. Released soluble sugar (reducing end) concentrations were analyzed by the dinitrosalicylic acid (DNS) method, as previously described [36]. Final soluble sugar concentrations were determined against a glucose calibration curve, and specific activity [µM reducing ends (µg protein)^−1^ min^−1^] was calculated.

### Proteolysis

The purified cellulosome samples were dissolved in 8 M urea in 100 mM ammonium bicarbonate, reduced by dithiothreitol at a final concentration of 2.8 mM (60 °C for 30 min) and modified with 8.8 mM iodoacetamide in 100 mM ammonium bicarbonate (30 min, room temperature, in the dark). The reduced, modified samples were then digested by modified trypsin (Promega, Madison, WI) at a 1:50 enzyme-to-substrate ratio in 2 M urea, 25 mM ammonium bicarbonate, overnight, followed by a second digestion step (4 h).

### Mass spectrometry analysis

Following the digestion step, the resultant peptide mixture was desalted, dried, and re-suspended in 0.1% formic acid. The peptides were resolved by reverse-phase chromatography on 0.075 × 180-mm fused silica capillaries (J&W) packed with Reprosil reversed phase material (Dr. Maisch GmbH, Germany). The peptides were eluted with a linear 60-min gradient of 5–28% acetonitrile with 0.1% formic acid, 5-min gradient of 28–95%, and 15 min at 95% acetonitrile with 0.1% formic acid in water, at a flow rate of 150 nL/min. MS analysis was performed by Q Exactive plus mass spectrometer (Thermo Scientific, Waltham, MA) in a positive mode using repetitively full MS scan followed by collision-induced dissociation (CID) of the ten most dominant ions selected from the first MS scan. The MS data were analyzed using MaxQuant v1.5.1.2 software (Cox and Mann [37]) versus the *C. thermocellum* DSM1313 section of the NCBI-nr database with 1% FDR, and further analyzed against the Carbohydrate-Active enzymes (CAZY) database. Due to the repetitive nature of some cellulosomal subunit sequences, we considered only proteins identified by at least one unique peptide. Data were statistically analyzed using Perseus v1.5.0.31 (part of the MaxQuant package). Intensities were normalized by the previously described intensity-based absolute quantification (iBAQ) method [38]. Average and standard deviations of duplicate samples of CB- and MCC-derived cellulosomes and triplicates of glucose-, alSG-, alCS-, and acCS-derived cellulosomes were analyzed.

## Results and discussion

### Purification of different cellulosomes

In order to investigate the influence of different carbon sources on the cellulosome composition and consequently on its activity, *C. thermocellum* strain DSM1313 was grown on cellobiose (CB), microcrystalline cellulose (MCC), alkaline-pretreated switchgrass (alSG), alkaline-pretreated corn stover (alCS), and dilute acid-pretreated corn stover (acCS). The latter lignocellulosic biomasses are representative of industrially relevant feedstocks. Both alkaline- and dilute acid-based pre-treatments are well established and common in the bioethanol field, designed to enrich the cellulosic fraction, and to increase accessibility of hydrolytic enzymes. The chemical composition of the different lignocellulosic biomasses is shown in Table 1. In nature, *C. thermocellum* hydrolyzes the cellulose into soluble cellobiose units, which in turn are actively taken up by the bacterium and further hydrolyzed into glucose units by a cell-associated β-glucosidase. Soluble glucose can be directly utilized and used as the sole carbon source by some strains of *C. thermocellum* only after a prolonged adaptation period [13, 29, 30]. In this study, such an adaptation period was used to generate cellulosomes from glucose-based growth media. Production of cellulosome samples was accomplished in triplicate for each of the six different carbon sources. The soluble cell-free cellulosomes were purified and analyzed for their cellulolytic activity and subunit composition.

**Table 1:** Chemical composition of lignocellulosic biomasses used in this work

	Abbreviation	Cellulose^a^ (%)	Hemicellulose^a^ (%)	Lignin^a^ (%)	Non-ligno cellulose fraction^a^ (%)
Untreated switchgrass	SG	37	28	18	17
Untreated corn stover	CS	36	27	20	17
Alkaline-pretreated switchgrass (alSG)	alSG	56	20	21	3
Alkaline-pretreated corn stover (alCS)	alCS	64	16	13	7
Dilute acid-pretreated corn stover (acCS)	acCS	60	5	30	5

^a^% dry matter

### Activity assays

In order to calculate specific activities [µM reducing ends (µg protein)^−1^ min^−1^], substrate degradation was quantified and total protein concentration in each sample was measured. MS analysis revealed that in addition to cellulosomal proteins, the “purified” high-molecular weight cellulosomal fraction also contained unrelated proteins, namely proteins without any known direct lignocellulolytic function (e.g., S-layer domain-containing proteins or flagellin domain-containing proteins). Consequently, the specific activity measured in a given sample would be biased by the presence of the unrelated proteins and would further lower the specific activity. In order to overcome this discrepancy, the relative content of cellulosomal proteins and non-cellulosomal enzymes in each sample was calculated using the MS data and used for calculations of the “true” specific activity.

**Fig. 1:**
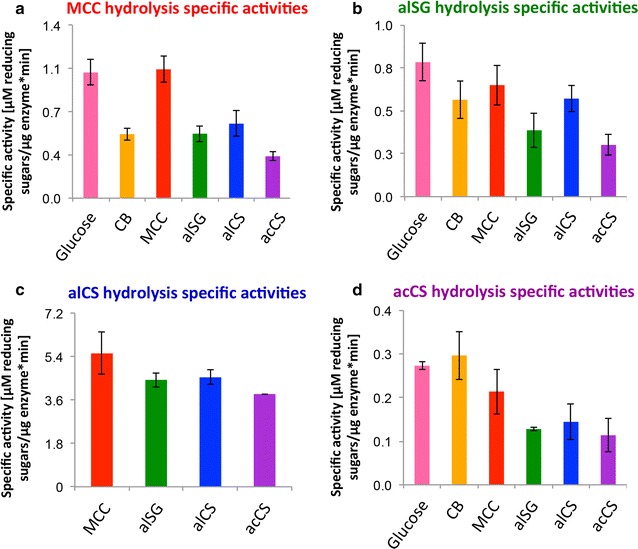
Specific activities of the various cellulosome fractions: cellulosomes derived from glucose-, cellobiose (CB)-, microcrystalline cellulose (MCC)-, alkaline-pretreated switchgrass (alSG)-, alkaline-pretreated corn stover (alCS)-, and dilute acid-pretreated corn stover (acCS)-based growth media were applied on the different substrates and released soluble sugars were measured by the DNS method. The percent content of relevant enzymes (cellulosomal subunits and soluble carbohydrate-active enzymes) in each sample was calculated using MS, and specific activities were calculated [µM reducing ends µg protein^−1^ min^−1^]. **a** Cellulosomes (20 µg) were applied to 70 mg MCC for 19 h; **b** cellulosomes (60 µg) were applied to 50 mg alSG for 16 h; **c** cellulosomes (3 µg) were applied to 50 mg alCS for 15 h. **d** Cellulosomes (50 µg) were applied to 50 mg acCS for 18 h. Cellulosomes and biomass dosages were determined in preliminary calibration assays in order to work in the near-linear range of the reaction (Additional file 2: Figure S2). All assays were performed at a final volume of 1 mL, at 70 °C, with the addition of 0.33 mg/mL equivalent of *Thermoanaerobacter brockii* β-glucosidase (CglT) in order to prevent feedback inhibition

Generally, the various isolated cellulosomes displayed varied specific activities on the different substrates, thus demonstrating the significant influence of the carbon source used in the growth media on the activity of the resulted cellulosome preparation (Fig. 1). One leading dogma assumes that cellulosomal subunit composition, generated from growth media supplemented with a specific carbon source, will lead to a superior activity towards that specific carbon source. For example, cellulosome preparations, generated from bacteria grown on corn stover, would be assumed to display relatively high hydrolytic activity towards the same substrate, compared to cellulosome preparations generated from wheat straw or switchgrass. The results obtained in this study do not entirely support this dogma. Although the specific activity of MCC-derived cellulosomes in hydrolyzing MCC was indeed higher than those of the other cellulosome preparations (Fig. 1), no advantage was found for the lignocellulosic biomass-derived cellulosome preparations in the hydrolysis of the same type of biomass used to generate them. Moreover, cellulosomes derived from glucose-based substrates (i.e., MCC, CB, and glucose itself) exhibited higher specific activities towards acCS, compared to cellulosomes from bacteria grown on lignocellulosic biomass, including acCS itself. In the same manner, glucose- and MCC-derived cellulosome preparations revealed higher specific activities towards hydrolysis of alSG compared to the alSG-derived cellulosome. Interestingly, glucose- and MCC-derived cellulosomes showed similar specific activities on all tested biomasses, including MCC.

Previous proteomic and transcriptomic studies have suggested that understanding the relationship between a specific carbon source and the resultant cellulosomal subunit composition will enable selection of key potent enzymes for efficient hydrolysis of specific substrates important for industry [25, 28, 39]. Conversely, the results reported here suggest that glucose- and MCC-derived cellulosomes are comparable or superior in their polysaccharase activity on all cellulosic substrates tested, to those obtained from cells grown on cellobiose or lignocellulosic substrates. Consequently, glucose- and MCC-derived cellulosomes are better sources for determining optimal compositions of key enzymes than those derived from the other substrates tested. The finding that differential assembly processes and consequent cellulosome compositions do not necessarily display more efficient cellulosomes is supported by similar findings of hydrolyzing pretreated switchgrass by *Clostridium clariflavum* cellulosomes and partially by the hydrolysis of untreated wheat straw by *Clostridium cellulolyticum* cellulosomes [40, 41].

Several explanations can be suggested in order to explain these findings. For example, an optimized tailored cellulosome, assembled by the bacterium to fit a carbon source, is not necessarily the most active one. Alternatively, controlled hydrolysis rate, and the resultant controlled soluble sugar release, may serve to avoid competition and/or interact with surrounding satellite microorganisms [42]. In the same manner, uncontrolled biomass degradation can increase the release of inhibitors. Furthermore, the hydrolysis rate has to be synchronized with the cellobiose uptake rate, since high cellobiose concentrations can inhibit cellulosome activity [43]. Thus, it is likely that differential assembly of cellulosomal subunits in response to carbon source is designed to regulate the hydrolysis rate rather to achieve the highest rate of hydrolysis. Another possible explanation lies in the nature of the biomass used in the original bacterial cell culture. Pure cellulose chains, as well as soluble cellobiose or glucose, are not common in nature (except for wastes derived from human society). Even the lignocellulosic biomasses used in the present study have undergone pre-treatment steps, as required for the bioethanol production process. Therefore, the carbon sources used in this study are ‘unnatural’ substrates (i.e., not available to the bacteria in nature). The bacterial regulation apparatus, designed to respond to the natural carbon source in the environment, might be ineffective or even a source of interference for degradation of the ‘unnatural’ cellulosic substrates used here. The resultant cellulosome preparation would thus show no advantage in their hydrolysis [41].

### Mass spectrometry analysis

The compositions of purified cellulosomes were analyzed by label-free LC–MS/MS. Peptide sequences from MS data were compared to the annotated protein database of *C. thermocellum* DSM1313 (NCBI, RefSeq NC017304.1) and further compared to the CAZY database for characterized carbohydrate-active enzymes (http://www.cazy.org/) [44]. Measured intensities were normalized using the intensity-based absolute quantification (iBAQ) method. Since mass spectrometry measurements depend not only on the concentration of each protein but also on its amino acid sequence, different proteins of the same concentration might reveal different total intensity measurements. The iBAQ method normalizes the results in a way that two different proteins with the same molar ratio will show similar iBAQ intensity, thus enabling internal comparison among samples (i.e., between different proteins in a given sample) [38].

### Scaffoldins

**Fig. 2:**
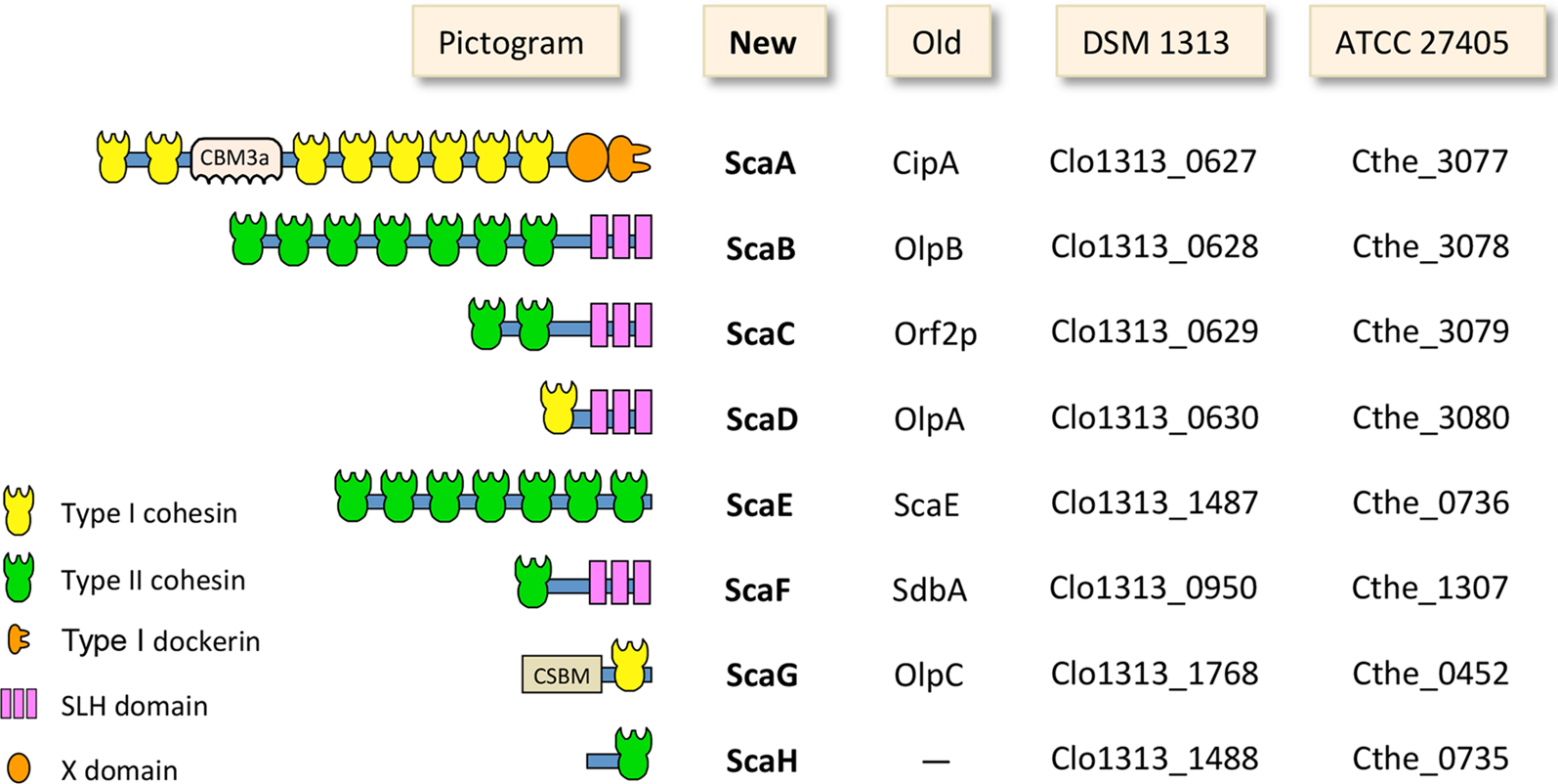
Schematic diagram of the eight *C. thermocellum* DSM1313 scaffoldins. The genome of *C. thermocellum* DSM1313 (RefSeq NC017304.1) was screened for cohesin-containing proteins, and annotated by NCBI annotation software. New (as reported in Bras et al. [45]) and old terminologies are shown for the scaffoldins, together with their gene loci in strain DSM 1313 and their equivalents in the ATCC 27405 strain. CBM, carbohydrate-binding module; SLH, surface-layer homology; CSBM, cell-surface binding module. The numbers of cohesins in ScaA and ScaB (8 and 7, respectively) shown in the figure are according to the recent report of Hong et al. [49]

Cellulosomes are heterogeneous sets of high-molecular weight multi-enzyme complexes composed of multi-domain structural scaffoldins and several dozen catalytic subunits. The binding of the catalytic subunits is mediated by the high-affinity noncovalent interactions between the multiple scaffoldin-borne cohesin modules and a dockerin module located in each catalytic subunit. The primary scaffoldin in *C. thermocellum,* CipA (Clo1313_0627), herein termed as ScaA (Fig. 2) according to Bras et al. [45], serves as a binding platform for the catalytic subunits via type I cohesin–dockerin interactions. In addition, *C. thermocellum* cellulosomes can be assembled into more complex cellulosomal suprastructures (polycellulosome complexes) via secondary scaffoldins. This type of assembly is mediated by the interaction of a type II dockerin module (located at the C terminus of the scaffoldin) with type II cohesins located in the secondary scaffoldin. Those secondary scaffoldins might be either cell-associated anchoring scaffoldins (mediating attachment of cellulosomes to the bacterial cell wall via their S-layer homology [SLH] domain [18, 19]) or soluble cell-free scaffoldins (lacking the SLH domain). Secondary scaffoldins, which bear several type II cohesins, can bind an equivalent number of primary scaffoldins and thus can assemble poly-cellulosomal complexes of different size, architecture, and content. The organization of the catalytic subunits on structural scaffoldins create a proximity effect that enhances synergy among neighboring enzymes [20, 46].

In order to analyze the structure of the different cellulosome samples, the genome of *C. thermocellum* DSM1313 (NC017304.1) [47] was screened for cohesin-bearing proteins. A schematic diagram of all *C. thermocellum* DSM1313 scaffoldins is shown in Fig. 2. Previously identified scaffoldins are now coined herein according to new terminology as reported in Bras et al. [45]. The scaffoldins of strain DSM1313 are similar in architecture and content to those of strain ATCC 27405 [48], with two noticeable differences: (A) according to the available genome sequence, DSM1313 ScaA contains six type I cohesin domains while ATCC 27405 ScaA (CipA) contains nine type I cohesin modules, and (B) the DSM1313 secondary scaffoldin ScaB contains four type II cohesin modules, whereas ATCC 27405 ScaB (OlpB) contains seven type II cohesin modules. While those discrepancies arise from sequence annotation, it is relevant to note that Hong et al. [49] have claimed that the genomic sequencing of the *cipA* and *olpB* genes in the DSM1313 strain was incorrect. Based on PCR reaction studies, these authors claimed that *C. thermocellum* DSM1313 ScaA contains eight cohesins (one less than that reported for *C. thermocellum* ATCC 27405 [50]) and ScaB contains seven cohesins (similar to that of *C. thermocellum* ATCC 27405 OlpB). In the present work, we have accepted the latter claims and used a value of eight type I cohesins for ScaA and seven type II cohesins for ScaB.

MS analysis revealed the presence of all eight known scaffoldins in all cellulosome samples, albeit some (i.e., ScaH and ScaE in some samples) were present only in relatively minor amounts.

### Secondary scaffoldins

**Fig. 3:**
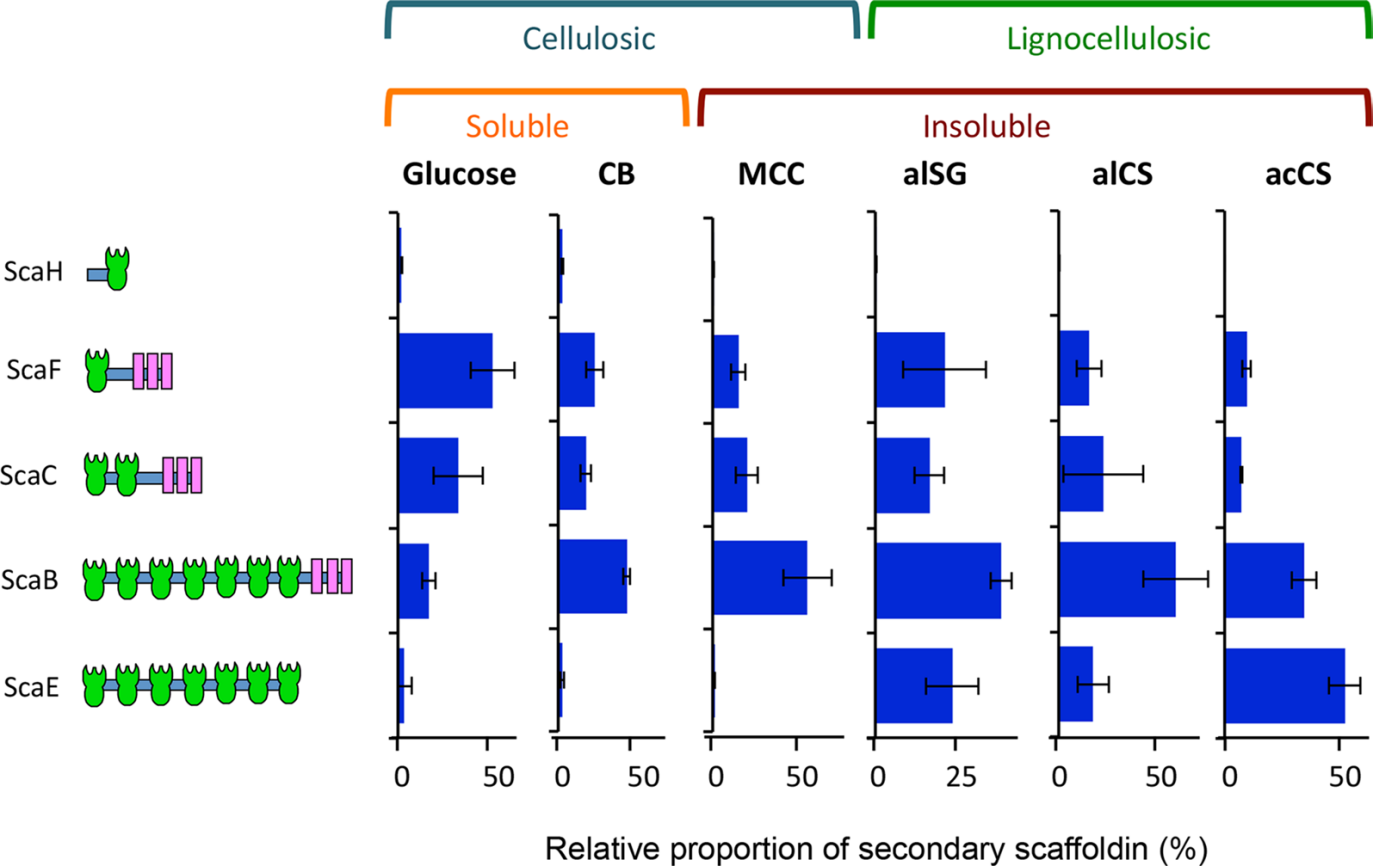
Differential distribution of secondary type II cohesin-containing scaffoldins. iBAQ intensities of all secondary scaffoldins in each sample were summed up, and the relative proportions of each secondary scaffoldin in each sample was calculated. The carbon source (see legend to Fig. 1 for definitions) used for the respective cellulosome production is shown in the graph

In order to shed light on the polycellulosomes suprastructures, the relative proportion of each secondary scaffoldin, derived from cells grown on each substrate, was assessed (Fig. 3). The different cellulosome samples exhibited differences in distribution of the secondary scaffoldins.

The most abundant secondary scaffoldin in most of the tested cellulosomal samples (i.e., CB-, MCC-, alSG-, and alCS-derived cellulosomes) was ScaB. In contrast, the most abundant secondary scaffoldin for acCS-derived cellulosomes was ScaE. Both ScaB and ScaE (containing seven cohesin models, respectively) enable the formation of large polycellulosome superstructures. Conversely, the most abundant secondary scaffoldin in the glucose-derived cellulosomes was ScaF (formerly SdbA), containing only one cohesin module. The combined amount of both ScaB and ScaE in the glucose-derived samples was only 19% (compared to 51–83% in the other samples). These results suggest the formation of significantly smaller cellulosomal complexes in the glucose-derived cellulosomes. It was therefore of interest to consider whether the multiple polycellulosome suprastructures assembled by the various secondary scaffoldins would directly influence their activity on (ligno) cellulosic substrates. For example, cellulosome architecture could possibly increase the synergistic effect by assembling several individual cellulosomes into a higher order structure, each of which would bear a different composition of catalytic subunits. In addition, the size of the polycellulosome complex may influence its accessibility to the crystalline cellulosic regions of the substrate [51]. However, no connection was observed in the present study between the distribution of secondary scaffoldins and the activity of the various cellulosome preparations. Intriguingly, glucose- and MCC-derived samples revealed similar levels of activity (Fig. 1), despite of the differences in the distribution of their secondary scaffoldins. In addition, CB- and MCC-derived cellulosomes exhibited similar patterns of secondary scaffoldins but significantly different specific activities when assayed for hydrolysis of MCC. These results indicate that the distribution of secondary scaffoldin per se does not significantly influence specific activity. Our results correspond to previously described reports, demonstrating that secondary scaffoldins exhibit only a minor effect on the hydrolysis of cellulosic substrates compared to the critical effect of ScaA [49, 52–55].

ScaE contains seven type II cohesins but lacks an SLH domain. The relative proportion of ScaE was higher (one order of magnitude) in lignocellulosic substrate-derived cellulosomes (i.e., alSG, alCS, and acCS) compared to that of cellulosomes derived from cells grown on pure homogeneous cellulose and its degradation products (i.e., MCC, CB, and glucose). ScaE was found to mediate the assembly of large polycellulosome complexes [52]. Those complexes were termed long-range, cell-free cellulosomes due to the lack of SLH or any other known module or sequence that would facilitate their binding to the *C. thermocellum* cell wall [52]. It was suggested that such “cell-free cellulosomes” can diffuse away from the cell and degrade polysaccharide substrates remotely from the bacterial cell [52]. It was further suggested that such a system can accommodate and target catalytic subunits involved in the hydrolysis of “non-cellulosic” [25] (i.e., hemicellulosic) polysaccharides via the CBM domain of the scaffoldin. In this context, the hemicellulase subunits would thus expose the cellulose microfibrils of complex lignocellulosic substrates, thereby enabling its sequential hydrolysis by the cell-surface cellulosomes. Interestingly, our results demonstrate relatively high portions of ScaE when *C. thermocellum* DSM1313 was grown on lignocellulosic substrates (where exposure of cellulose fibrils is necessary) and relatively low portions of ScaE when *C. thermocellum* DSM1313 was grown on pure cellulose or its degradation products (where such exposure of the microfibrils is not required).

Another “cell free” secondary scaffoldin produced by this bacterium is ScaH (that contains only a single type II cohesin without an SLH domain). This secondary scaffoldin was the least abundant in all samples. Nevertheless, the relative portion of ScaH in cellulosomes derived from soluble substrates (i.e., CB and glucose) was one order of magnitude higher compared to those of cellulosomes derived from the lignocellulosic substrates (i.e., alSG, alCS, and acCS) and ~2.5-fold higher than that of MCC-derived cellulosome.

### Primary scaffoldins

Among all structural proteins, the relative proportion of primary scaffoldins (type I cohesin-containing proteins) was larger than that of the secondary scaffoldins in all samples, accounting for 58–63% in the glucose-, CB-, and MCC-derived cellulosomes, and 76–80% for the lignocellulose-derived cellulosomes (Table 2).

**Table 2:** Relative proportion of primary and secondary scaffoldins in the different samples

Carbon source	Primary scaffodins (%)	Secondary scaffoldins (%)
Glucose	60 ± 3	40 ± 3
CB	63 ± 1	37 ± 1
MCC	58 ± 6	42 ± 6
alSG	76 ± 2	24 ± 2
alCS	78 ± 6	22 ± 6
acCS	80 ± 2	20 ± 2

**Fig. 4:**
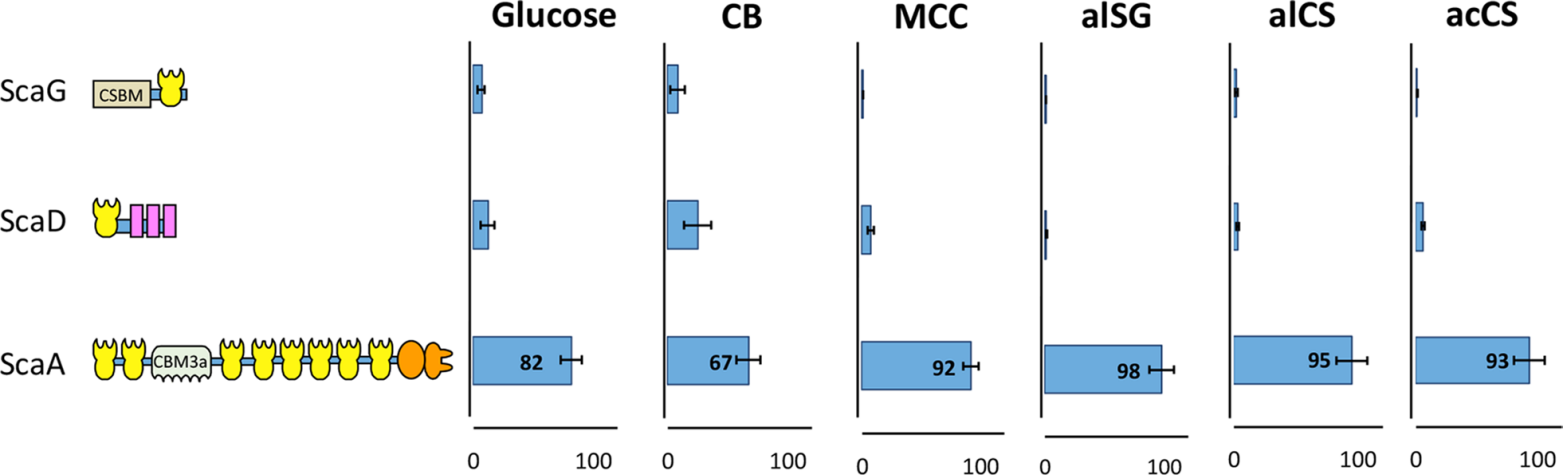
Differential distribution of primary (type I cohesin-containing) scaffoldins. iBAQ intensities of the primary scaffoldins in each sample were summed up, and the relative proportions of each primary scaffoldin in each sample was calculated. The carbon source (see legend to Fig. 1 for definitions) used for the respective cellulosome production is shown in the graph

Figure 4 summarizes the distribution of the primary scaffoldins. ScaA, by far, comprised the great majority of the primary scaffoldins, accounting for 67% (in CB-derived cellulosomes) up to 98% (in alSG-derived cellulosomes), followed by ScaD, and ScaG. The relative proportion of ScaD and ScaG (single type I cohesin) was significantly lower in the insoluble substrate-derived cellulosomes (7.7, 2.3, 5.1, and 6.5% for MCC-, alSG-, alCS-, and acCS-derived cellulosomes, respectively) compared to that of the glucose- and cellobiose-derived cellulosomes (18.3 and 33% for glucose- and CB-derived cellulosomes, respectively). These results correlate with the measured intensities of Cthe_0452 (ScaG homolog) in the ATCC 27405 CB-derived cellulosomes versus those derived from insoluble substrates [25].

### Occupancy of type II cohesins

ScaA is the only primary scaffoldin that contains a type II dockerin that can bind to the type II cohesins of secondary scaffoldins. Comparison of the amount of ScaA to that of the available type II cohesins can also provide insight into the suprastructure of the different cellulosome samples. In order to calculate the type II dockerin-to-cohesin ratio, the relative portions of the secondary scaffoldins were multiplied by the number of their cohesins, and the ScaA/type II cohesin ratio was calculated. The ratio for CB- and MCC-derived cellulosomes was 0.26 ± 0.0 and 0.27 ± 0.07, respectively. Significantly higher ratios were found for the lignocellulosic biomass-derived cellulosomes (0.61 ± 0.03, 0.65 ± 0.2, and 0.63 ± 0.09 for alSG-, alCS-, and acCS-derived cellulosomes, respectively), thus indicating a higher degree of occupancy and complexity in the lignocellulosic biomass-derived cellulosomes. Interestingly, cellulosomes that exhibited higher occupancies for type II cohesins (i.e., lignocellulosic substrate-derived cellulosomes) generally showed relatively lower levels of specific activities (Fig. 1). Surprisingly, the ratio of glucose-derived cellulosomes was 0.51 ± 0.09, similar to those of the lignocellulosic substrate-derived cellulosomes. Yet, the glucose-derived cellulosomes exhibited relatively high specific activity values. This result may be related to the distribution of secondary scaffoldins in the glucose-derived cellulosomes, which shows a majority of the monovalent scaffoldin ScaF, as opposed to the other types of cellulosome. Thus, in this case, the degree of occupancy may only have a negligible influence on activity. Incomplete occupancy of type II cohesins was previously reported for *C. thermocellum* ATCC 27405 cellulosomes, with even lower occupancy levels at the proteome [25] and transcriptome levels [56].

### Cellulosomal subunits

In order to analyze subunit composition of the different cellulosome samples, the *C. thermocellum* DSM1313 genome was screened for type I dockerin-containing proteins. Seventy-five hypothetical cellulosomal subunits were thus revealed. Altogether, the proteomic study revealed 67 different dockerin-containing proteins in the cellulosome samples. For comparison among the different cellulosome samples, iBAQ intensities of cellulosomal subunits of a given sample were normalized with that of the primary scaffoldin ScaA (CipA) from the same sample, thus creating a relative abundance index. The use of ScaA as an internal standard provides information regarding the composition of the cellulosome subpopulations within each sample (as described in previous proteomic studies [25, 26]). The values obtained reveal differences between samples, by comparing the relative abundance of the individual subunits. It is important to emphasize that ScaA is a glycosylated protein [57–60]. Glycosylation changes the molecular weight of a peptide, which interferes with its identification by the LC–MS/MS. Since iBAQ normalization takes into account the number of theoretical peptides, the iBAQ intensity of ScaA is probably underestimated. Nevertheless, the relative abundance index enables comparison *within* samples, and, assuming identical glycosylation of ScaA in all samples, *among* samples as well [25].

**Fig. 5:**
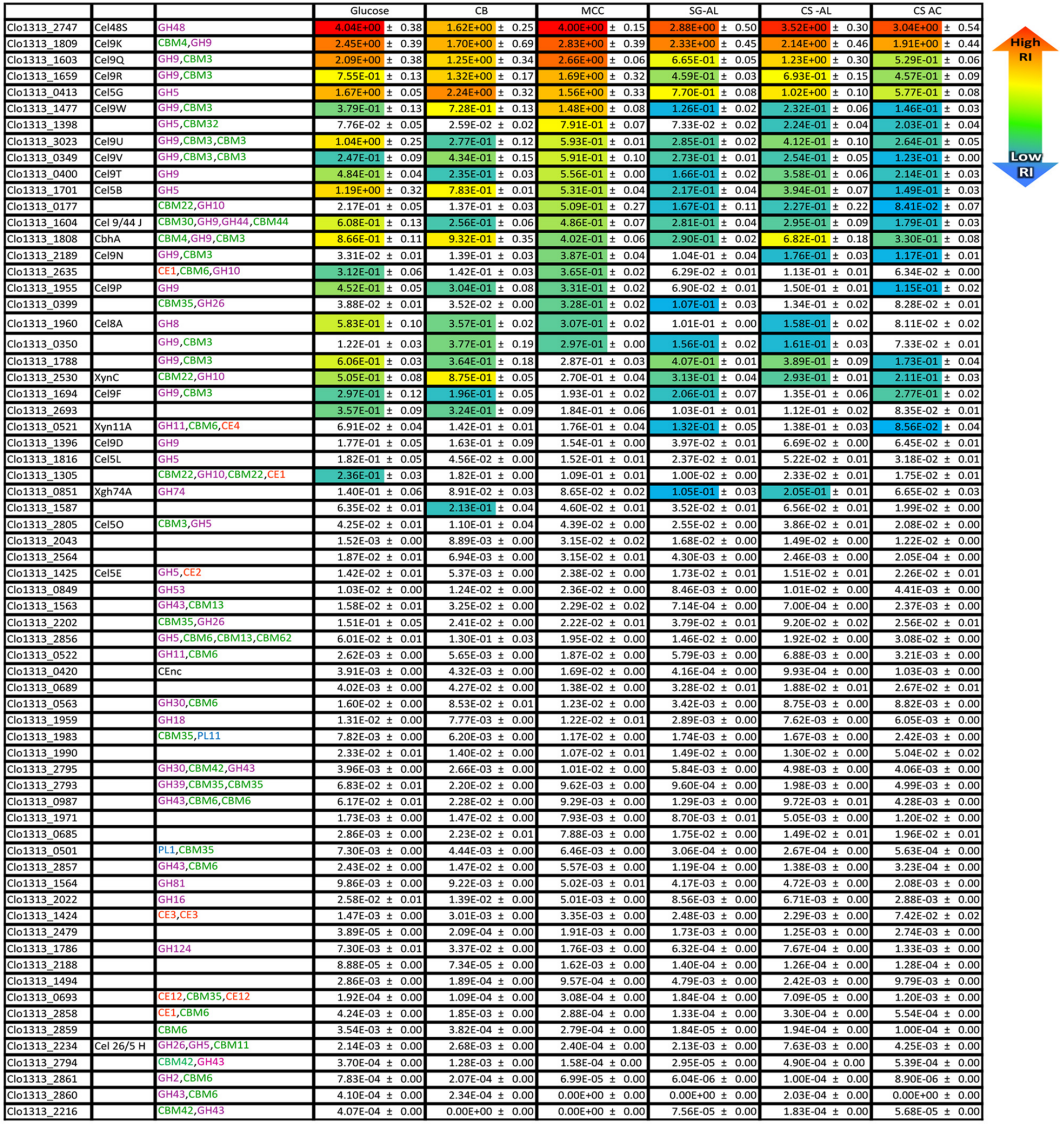
Cellulosomal subunit composition. Subunit compositions of the different cellulosomes were analyzed by label-free LC–MS/MS mass spectrometry. The intensities were normalized by intensity-based absolute quantification (iBAQ) method. The resultant iBAQ intensities of type I dockerin-containing subunits were divided by the iBAQ intensity of ScaA in each sample, thereby generating a relative abundance index. Average and standard deviations of duplicate samples of CB- and MCC-derived cellulosomes and triplicates of glucose-, alSG-, alCS-, and acCS-derived cellulosomes were analyzed. The 20 most abundant subunits in each sample were rated by a colored scale. Gene ID and CAZY annotation of the subunits are mentioned in the table too. GH, glycoside hydrolase; CBM, carbohydrate-binding module; CE, carbohydrate esterase

**Fig. 6:**
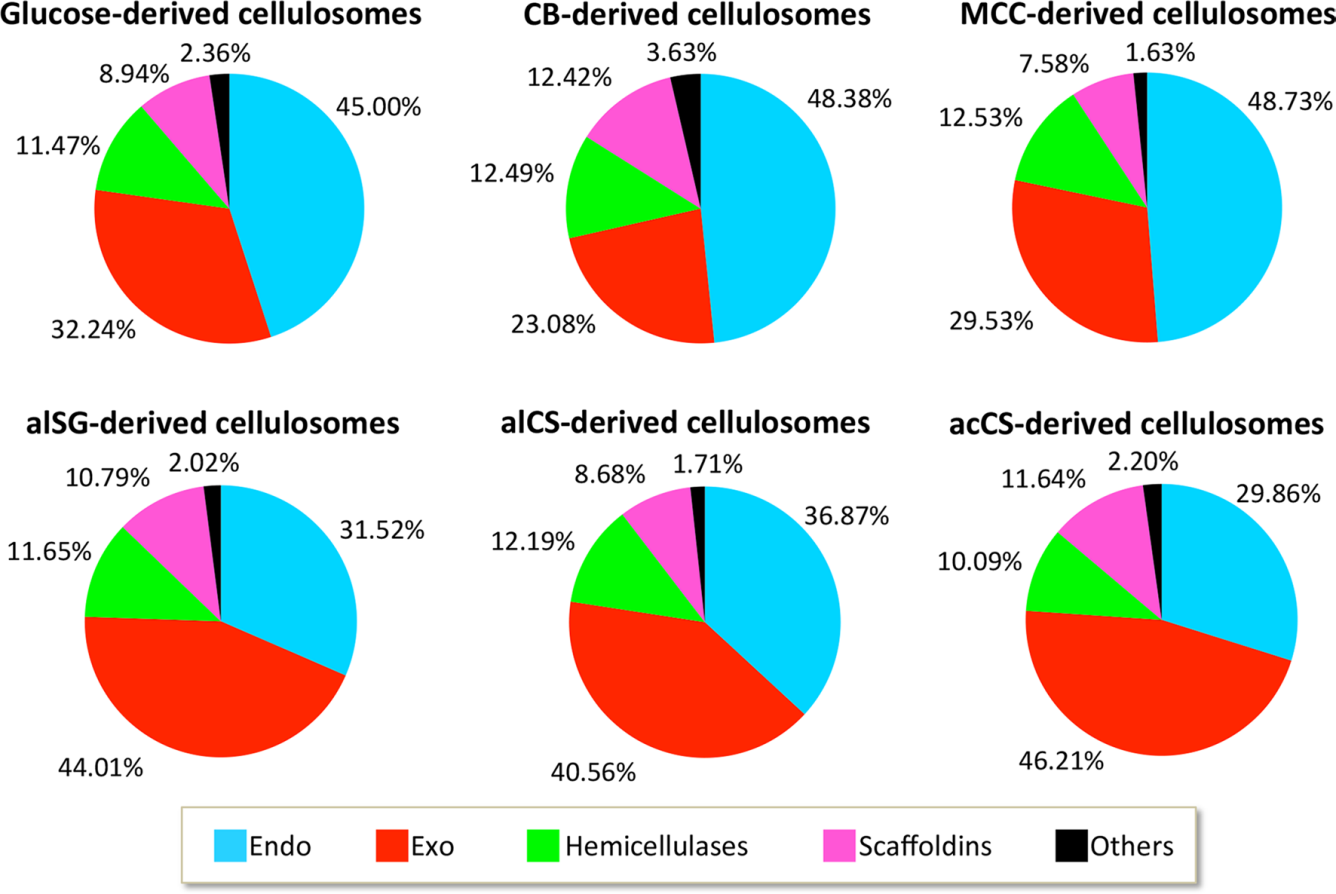
Cellulosomal subunit distribution: Relative abundance of all type I dockerin-containing subunits were summed up and defined as 100%. The subunits were divided into five groups according to their activity as detailed in Additional file 3: Table S1. The relative proportion of each group was calculated. Blue, endoglucanase; red, exoglucanase; green, hemicellulase; pink, scaffoldins; and black, others

The table in Fig. 5 summarizes the relative abundance values of the cellulosomal subunits detected in the different samples. The twenty most abundant enzymes are marked and rated using the color scale shown in the table. The five most abundant proteins accounted for at least 50% of total cellulosomal subunits in each sample, with a higher percentage (60–64%) in the lignocellulosic biomass-derived cellulosomes, compared to those (50–56%) in the glucose-, cellobiose-, and MCC-derived cellulosomes. The most abundant subunits in all samples included exoglucanases Cel48S (Clo1313_2747) and Cel9K (Clo1313_1809), and endoglucanases Cel9Q (Clo1313_1603), Cel9R (Clo1313_16590), and Cel5G (Clo1313_0413), indicating their importance in biomass degradation. Out of the 20 most abundant catalytic subunits, 12 were common among all samples. The detected cellulosomal subunits were analyzed for their functional class distribution (Fig. 6; Additional file 3: Table S1). The cellulase (endo- and exoglucanase) portion was about 76–78% of the total cellulosomal subunits in all samples, except for the CB-derived samples, in which the cellulase portion was somewhat lower (71%). The endoglucanase-to-exoglucanase ratio was found to be higher in glucose-, cellobiose-, and MCC-derived cellulosomes (1.4, 2, and 1.65, respectively), compared to that of the lignocellulosic biomass-derived cellulosomes (0.71, 0.9 and 0.64 for alSG-, alCS-, and acCS-derived cellulosomes, respectively). Since glucose and MCC-derived cellulosomes showed higher specific activity, we may hypnotize that an endo-to-exo ratio of ~1.5 is recommended for assembly of cellulolytic cocktails based on *C. thermocellum* enzymes.

### Exoglucanases

Excluding CB-derived cellulosomes, all samples revealed a similar pattern of exoglucanases, in which the relative abundance value of Cel48S was the highest, cell9K showed the second highest, followed by CbhA, and finally Cel5O with the lowest level of abundance. In the CB-derived cellulosomes, Cel9K showed the highest relative abundance among the four exoglucanases. Cel48S and CbhA showed lower levels, but the differences were not statistically significant. Similar trends of lower Cel48S abundance in the CB-derived cellulosomes compared to that of MCC-derived cellulosomes was previously reported for *C. thermocellum* ATCC 27405, both at the transcriptome and protein levels [21, 25, 61, 62], thus confirming our present data. The lignocellulosic biomass-derived cellulosomes revealed modest decreases in the relative abundance of Cel48S, compared to that of the MCC-derived cellulosomes. Only glucose-derived cellulosomes revealed a similar level of relative abundance of Cel48S to those of MCC-derived cellulosomes.

Cel9K showed similarly, higher relative abundance in the MCC- and glucose-derived cellulosomes, and the lowest level in CB-derived cellulosomes. Nevertheless, these differences were not statistically significant (A similar pattern was reported for *C. thermocellum* ATCC 27450 [26]). A different pattern was found for CbhA; however, which revealed higher relative abundance in the CB- and glucose-derived cellulosomes compared to the MCC- and the lignocellulosic biomass-derived cellulosomes. Cel9 K and CbhA are tandem genes, containing a similar type of modular structure (except the lack of X1-like domains and the CBM3 domain in Cel9K), with 94% identity among the modules, and therefore suggested to arose by gene duplication [63]. Due to the similarity in enzyme structure, it would be tempting to assume a similar function. Thus, the non-similar abundance pattern of the two proteins was somewhat surprising. The different role of CbhA and Cel9 K remains to be explored.

### Endoglucanases

Among the different cellulosomal samples examined in this work, the twenty most abundant catalytic endoglucanase subunits always included the following enzymes: Cel9Q, Cel9R, Cel5G, Cel9W, Cel9U, Cel9V, Cel9T, Cel5B, and Cel9/44J. In recent preliminary data (Additional file 4: Table S2), Cel9/44J and Cel9D exhibited the highest endoglucanase activity of 25 *C. thermocellum* cellulosomal subunits examined. Surprisingly, Cel9D showed low relative abundance and therefore did not appear in the latter list in any of the analyzed samples. The data support the above claim that the cellulosomal subunit composition, derived from cells grown on the different substrates, is not necessarily optimized for efficient substrate hydrolysis.

### Type I dockerin-to-ScaA ratio

Type I dockerin-to-ScaA (type I dockerin/scaffoldin) ratio was calculated by summing up the relative abundance values for all of the type I dockerin-containing subunits in each sample. The ratio was almost double in the MCC- and glucose-derived cellulosomes (22.7 and 20.9 molecules per ScaA, respectively) compared to those of the alSG- and acCS-derived cellulosomes (11.2 and 10.1 molecules per ScaA, respectively) and higher than the alCS- and CB-derived cellulosomes (14.3 and 16.5 molecules per ScaA, respectively). The results revealed higher numbers of cellulosomal subunits per scaffoldin in the MCC- and glucose-derived cellulosomes, which might thus explain the observed higher hydrolytic activity of the latter cellulosomes.

### Soluble carbohydrate-active enzymes

Besides cellulosomal (dockerin-containing) enzymes, *C. thermocellum* produces a repertoire of soluble carbohydrate-active enzymes without dockerins as well. In order to characterize these non-cellulosomal enzymes and their relative content in the different samples, MS results were analyzed and compared to the *C. thermocellum* DSM1313 CAZY database (for GH/PL/GT/CE/CBM containing enzymes). In total, 28 additional non-cellulosomal carbohydrate-active enzymes were identified in the various cellulosome samples (Additional file 5: Table S3). The presence of un-complexed subunits was also supported by the detection of the structural scaffoldin ScaG. The present results are in line with previously described reports for *C. clariflavum* cellulosomes [40]. The detection of soluble carbohydrate-active enzymes in the high-molecular-weight fractions may reflect the result of non-specific interactions or unknown specific interactions that are not mediated by cohesin-dockerin interactions.

### Unique expression patterns

Three cellulosomal subunits were significantly upregulated in cellulosomes derived from the defined cellulosic substrates, that is, glucose-, cellobiose-, and MCC-derived cellulosomes: Clo1313_1305 (CBM22-GH10-CBM22-dockerin-CE1), Clo1313_1563 (GH43-CBM13-dockerin), and Clo1313_0501 (PL1-dockerin-CBM35). Comparison of cellulosomes derived from the soluble substrates (i.e., cellobiose and glucose) versus those from the insoluble substrates revealed significant upregulation of the non-cellulosomal protein Clo1313_0397 (SLH-CBM54-GH16-CBM4-CBM4-CBM4-CBM4-CBM4) and the cellulosomal subunit Clo1313_2857 (GH43-CBM6-dockerin). The cellulosomal subunit Clo1313_2479 (containing no identified CAZy module) was significantly downregulated. The role of these enzymes is as yet unknown.

### Comparison of *C. thermocellum* strains

The ‘classic’ reference, *C. thermocellum* strain ATCC 27405, was intensively studied in the past at the proteomic and transcriptomic level, while growing the cells on several different carbon sources [21, 25, 28, 39]. A recent release of the complete *C. thermocellum* strain DSM 1313 genome sequence [47] enabled differential omic studies, including both transcriptome and proteome. Moreover, the only successful gene-directed mutagenesis has been reported for strain DSM 1313, while strain ATCC 27405 is not easily amenable to genetic manipulations [64]. The composition of MCC-derived cellulosomes from strain DSM1313 reported here showed differences in subunit composition compared to that previously described for equivalent preparations from strain ATCC 27405. At least nine undetected cellulosomal enzymes in strain ATCC 27405 cellulosome [25] were detected in this study using the DSM 1313 strain: Clo1313_3023 (Cel9U, which was also detected earlier by Zverlov et al. [65]), Clo1313_2793 (GH39), Clo1313_1564 (GH81), Clo1313_0501 (PL10, Clo1313_2794, Clo1313_2858 (CE1), Clo1313_2859 (GH141), Clo1313_2860 (GH43), and Clo1313_2861 (GH2) (Fig. 5). The last five proteins showed relatively low but measurable relative abundance. In this context, Clo1313_2858, Clo1313_2859, Clo1313_2860, and Clo1313_2861 are located on the same operon. The orthologous operon in strain ATCC 27405 was found to be disrupted by a putative 2419 bp insertion sequence element located within the 5′ end of the Clo1313_2861 orthologue (annotated as two different genes: Cthe_2197 and Cthe2200). Such an insertion sequence does not exist in the DSM 1313 strain. The most abundant endoglucanases in the DSM1313 cellulosome, i.e., Cel9Q, Cel9R, Cel5G, and Cel9W, showed 3.7-fold to fivefold higher relative abundance, compared to the strain ATCC 27405-derived cellulosome [25]. In contrast, Cel8A, and Cel5E showed relatively lower abundance (fivefold and tenfold, respectively) in the DSM1313 cellulosomes, compared to those of strain ATCC 27405. Xyn11A (Clo1313_0521), which was the third most abundant protein in the ATCC 27405 cellulosome system, showed fivefold lower relative abundance in the DSM1313 system. We assume that higher expression of the Xyn11A homologue in ATCC 27405 (Cthe_2972) might be explained by a different gene organization in the two *C. thermocellum* genomes. While the *xyn11A* gene of DSM 1313 is a second ORF of a two-cistronic operon (*xyn11B*/Clo1313_0522-*xyn11A*/Clo1313_0521), the ATCC 27405 genome has only one gene, *xyn11A*, while a *xyn11B* homologue appears to be omitted from this genome.

## Conclusions

Our results demonstrate that cells grown on a given lignocellulosic substrate do not necessarily produce cellulosomes that exhibit enhanced activity levels on that particular substrate. Surprisingly, MCC- and glucose-derived cellulosomes showed superior performance even towards degradation of complex lignocellulosic substrates. The latter cellulosome preparations exhibited distinctive characteristics, e.g., elevated endoglucanase-to-exoglucanase ratios and enzyme-versus-ScaA ratios. The most abundant subunits in all tested cellulosomes included Cel48S, Cel9K, Cel9Q, Cel9R, and Cel5G, indicating their preferential contribution and importance to deconstruction of complex cellulosic substrates. Our results should be implemented in the future for fabrication of efficient designer cellulosomes, for formulation of recombinant cellulolytic cocktails based on *C. thermocellum* enzymes or for engineering *C. thermocellum* strains with improved lignocellulosic biomass-converting abilities.

## Additional files

**Additional file 1: Figure S1.** Purification profile of the different cellulosomes by Gel filtration chromatography. *C. thermocellum* growth media were centrifuged (10,900 g, 7 min), and the supernatant fluids were carefully removed from the pellet and concentrated 40 times using a Pellicon XL biomax 300 cassette (Millipore, Cat. No. PXB300C50). Concentrated samples were fractionated by size exclusion chromatography using a SuperdexS-200 prep grade 16/60 gel filtration column (GE Healthcare). (A) Chromatogram of glucose-derived cellulosomes (B) Chromatogram of CB-derived cellulosomes (C) Chromatogram of MCC-derived cellulosomes (D) Chromatogram of alSG-derived cellulosomes (E) Chromatogram of alCS-derived cellulosomes (F) Chromatogram of acCS-derived cellulosomes. Fractions containing the cellulosomes (according to SDS-PAGE analysis) are marked by black arrows. All cellulosomes-containing fractions were eluted immediately after void value due to the separation range of the column.

**Additional file 2: Figure S2.** Calibration of the near-linear range of the various substrate hydrolyses. Increased cellulosome dosages were applied on (**A**) 7% microcrystalline cellulose [MCC], (**B**) 5% alkaline-pretreated switch grass [alSG], (**C**) 5% alkaline-pretreated corn stover [alCS] and (**D**) 5% dilute acid-pretreated corn stover [acCS], and the samples were incubated overnight at 70 °C. Released sugar concentrations were measured by *dinitro*salicylic acid (DNS) method, as previously described [36]. All assays were performed with the addition of 0.33 mg/ml equivalent of *Thermoanaerobacter brockii* β-glucosidase (CglT) in order to prevent feedback inhibition. Enzyme loadings of 20, 50, 3 and 50 µg/ml for MCC, alSG, alCS and acCS hydrolysis assays, respectively, were chosen for activity measurements (Black arrows).

**Additional file 3: Table S1.** Functional classes distribution of *C. thermocellum* DSM 1313 cellulosomal subunits. Cellulosomal subunits detected in the *C. themocellum D*SM 1313 proteome were sorted into five functional classes: endoglucanases, exoglucanases, hemicellulases, scaffoldins and others.

**Additional file 4: Table S2.** Activities of *C. thermocellum* cellulosomal enzymes. Recombinant *C. themocellum* cellulosomal (type I dockerin-containing) enzymes were a kind gift of CelDezyner Ltd. (Rehovot, Israel). Activity assays were conducted in a final volume of 1 ml, containing 50 mM acetate buffer, 1% carboxymethyl cellulose (sodium salt, low viscosity CMC, BDH chemicals) and 7 nM enzyme. Samples were incubated with shaking for 3 h at 60 °C. Released soluble sugar (reducing ends) concentrations were analyzed by the dinitrosalicylic acid (DNS) method, as previously described [36]. Final soluble sugar concentrations were determined against a glucose calibration curve, and CMCase activities [µM reducing ends·µmol enzyme^−1^·min^−1^] were calculated. *A thermostable clone of Cel8A [66] was used.

**Additional file 5: Table S3.** Soluble (non-cellulosomal) carbohydrate-active enzyme composition. Soluble (dockerin-lacking) carbohydrate-active enzyme compositions of the different cellulosomes were analyzed by label-free LC–MS/MS mass spectrometry. The intensities were normalized by the intensity-based absolute quantification (iBAQ) method. The resultant iBAQ intensities were divided by the iBAQ intensity of ScaA in each sample, thereby generating a relative abundance index. Standard deviations of duplicate samples of CB- and MCC-derived cellulosomes and triplicates of glucose-, alSG-, alCS-, and acCS-derived cellulosomes were analyzed. Gene ID and CAZy annotation of the subunits are designated. Acronyms: GH, glycoside hydrolase; CBM, carbohydrate-binding module; CE, carbohydrate esterase; GT, glycosyl transferase.

## Abbreviations

CB: cellobiose
MCC: microcrystalline cellulose
alSG: alkaline-pretreated switch grass
alCS: alkaline-pretreated corn stover
acCS: dilute acid-pretreated corn stover
MS: mass spectrometry
iBAQ: intensity-based absolute quantification
CBM: carbohydrate-binding module
GH: glycoside hydrolase
CE: carbohydrate esterase
GT: glycosyl transferase
PL: polysaccharide lyase
Sca: scaffoldin
SLH: S-layer homology
Xyn: xylanase
SDS-PAGE: sodium dodecyl sulfate polyacrylamide gel electrophoresis
