# Supplementary material for: How does cellulosome composition influence deconstruction of lignocellulosic substrates in Clostridium (Ruminiclostridium) thermocellum DSM 1313?
Source: Biotechnol Biofuels. 2017 Sep 18;10:222. doi: 10.1186/s13068-017-0909-7 (PMC5604425; doi:10.1186/s13068-017-0909-7)
Supplement: Supplementary file 1 — Additional file 1: Figure S1. Purification profile of the different cellulosomes by Gel filtration chromatography. C. thermocellum growth media were centrifuged (10,900 g, 7 min), and the supernatant fluids were carefully removed from the pellet and concentrated 40 times using a Pellicon XL biomax 300 cassette (Millipore, Cat. No. PXB300C50). Concentrated samples were fractionated by size exclusion chromatography using a SuperdexS-200 prep grade 16/60 gel filtration column (GE Healthcare). (A) Chromatogram of glucose-derived cellulosomes (B) Chromatogram of CB-derived cellulosomes (C) Chromatogram of MCC-derived cellulosomes (D) Chromatogram of alSG-derived cellulosomes (E) Chromatogram of alCS-derived cellulosomes (F) Chromatogram of acCS-derived cellulosomes. Fractions containing the cellulosomes (according to SDS-PAGE analysis) are marked by black arrows. All cellulosomes-containing fractions were eluted immediately after void value due to the separation range of the column. [file 13068_2017_909_MOESM1_ESM.pdf]

Additional file 1

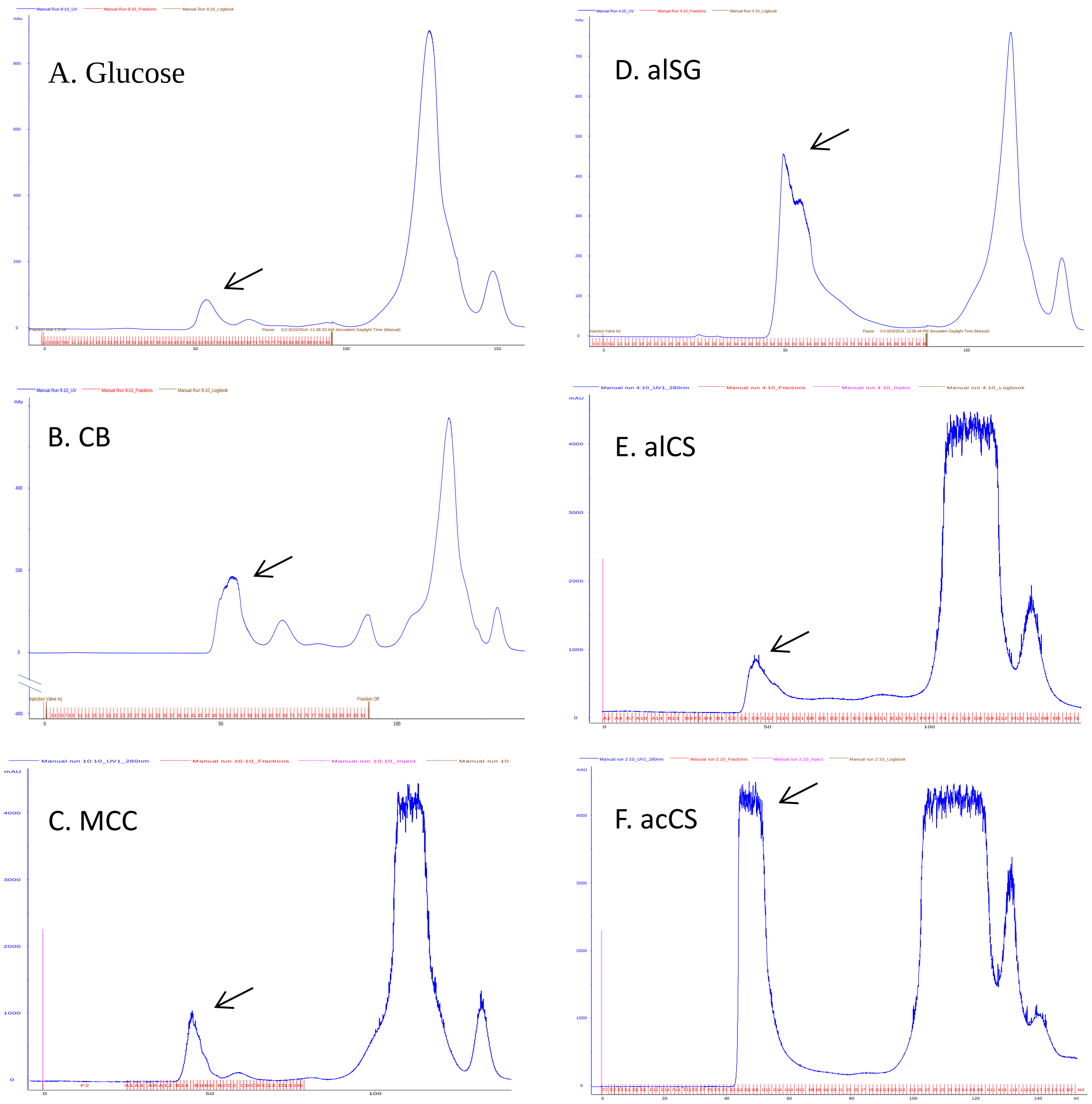

Figure S1. Purification profile of the different cellulosomes by gel filtration chromatography. *C. thermocellum* growth media were centrifuged (10,900 g, 7 min), and the supernatant fluids were carefully removed from the pellet and concentrated 40 times using a Pellicon XL Biomax 300 Cassette (Millipore, Cat. No. PXB300C50). Concentrated samples were fractionated by size exclusion chromatography using a HiLoad 16/600 Superdex 200 prep grade gel filtration column (GE Healthcare). (A) Chromatogram of glucose-derived cellulosomes (B) Chromatogram of CB-derived cellulosomes (C) Chromatogram of MCC-derived cellulosomes (D) Chromatogram of alSG-derived cellulosomes (E) Chromatogram of alCS-derived cellulosomes (F) Chromatogram of acCS-derived cellulosomes. Fractions containing the cellulosomes (according to SDS-PAGE analysis) are marked by black arrows. All cellulosome-containing fractions eluted immediately after the void volume, due to the separation range of the column.
