# Supplementary material for: How does cellulosome composition influence deconstruction of lignocellulosic substrates in Clostridium (Ruminiclostridium) thermocellum DSM 1313?
Source: Biotechnol Biofuels. 2017 Sep 18;10:222. doi: 10.1186/s13068-017-0909-7 (PMC5604425; doi:10.1186/s13068-017-0909-7)
Supplement: Supplementary file 2 — Additional file 2: Figure S2. Calibration of the near-linear range of the various substrate hydrolyses. Increased cellulosome dosages were applied on (A) 7% microcrystalline cellulose [MCC], (B) 5% alkaline-pretreated switch grass [alSG], (C) 5% alkaline-pretreated corn stover [alCS] and (D) 5% dilute acid-pretreated corn stover [acCS], and the samples were incubated overnight at 70 °C. Released sugar concentrations were measured by dinitrosalicylic acid (DNS) method, as previously described [36]. All assays were performed with the addition of 0.33 mg/ml equivalent of Thermoanaerobacter brockii β-glucosidase (CglT) in order to prevent feedback inhibition. Enzyme loadings of 20, 50, 3 and 50 µg/ml for MCC, alSG, alCS and acCS hydrolysis assays, respectively, were chosen for activity measurements (Black arrows). [file 13068_2017_909_MOESM2_ESM.pdf]

Additional file 2

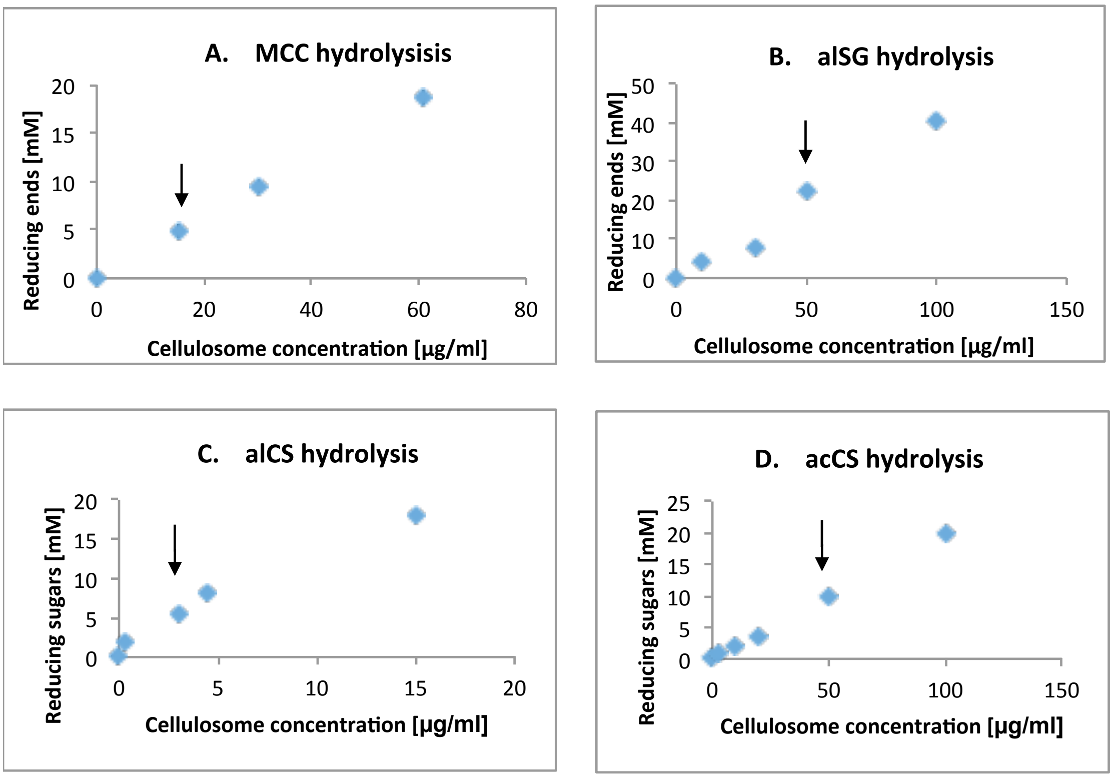

Figure S2. Calibration of the near-linear range of the various substrate hydrolyses. Increased cellulosome dosages were applied on **(A)** 7% microcrystalline cellulose (MCC), **(B)** 5% alkaline-pretreated switch grass [alSG], (**C**) 5% alkaline-pretreated corn stover [alCS] and (**D**) 5% dilute acid-pretreated corn stover [acCS], and the samples were incubated overnight at 70°C. Released sugar concentrations were measured by dinitrosalicylic acid (DNS) method, as previously described [1]. All assays were performed with the addition of 0.33 mg/ml equivalent of *Thermoanaerobacter brockii* β-glucosidase (CglT) in order to prevent feedback inhibition. Enzyme loadings of 20, 50, 3 and 50 µg/ml for MCC, alSG, alCS and acCS hydrolysis assays, respectively, were chosen for activity measurements (Black arrows).

References

1. Miller GL. Use of dinitrosalicylic acid reagent for determination of reducing sugar. Anal Biochem. 1959;31:426–428.
