# Supplementary material for: How does cellulosome composition influence deconstruction of lignocellulosic substrates in Clostridium (Ruminiclostridium) thermocellum DSM 1313?
Source: Biotechnol Biofuels. 2017 Sep 18;10:222. doi: 10.1186/s13068-017-0909-7 (PMC5604425; doi:10.1186/s13068-017-0909-7)
Supplement: Supplementary file 3 — Additional file 3: Table S1. Functional classes distribution of C. thermocellum DSM 1313 cellulosomal subunits. Cellulosomal subunits detected in the C. themocellum DSM 1313 proteome were sorted into five functional classes: endoglucanases, exoglucanases, hemicellulases, scaffoldins and others. [file 13068_2017_909_MOESM3_ESM.pdf]

Additional file 3

Table S1. Functional classes distribution of *C. thermocellum* DSM 1313 cellulosomal subunits Cellulosomal subunits detected in the *C. themocellum D*SM 1313 proteome were sorted into five functional classes: endoglucanases, exoglucanases, hemicellulases, scaffoldins and others.

Endoglucanases	Exoglucanases	Hemicellulases	Scaffoldins	Others
Clo1313_1603	Clo1313_2747	Clo1313_2216	Clo1313_1487	Clo1313_2693
Clo1313_1659	Clo1313_1809	Clo1313_2202	Clo1313_0628	Clo1313_1587
Clo1313_0413	Clo1313_1808	Clo1313_1788	Clo1313_0950	Clo1313_2043
Clo1313_1477	Clo1313_2805	Clo1313_1563	Clo1313_0629	Clo1313_2564
Clo1313_3023		Clo1313_1424	Clo1313_1488	Clo1313_0420
Clo1313_0349		Clo1313_1398	Clo1313_0627	Clo1313_0689
Clo1313_0400		Clo1313_1305	Clo1313_0630	Clo1313_1959
Clo1313_1701		Clo1313_0987	Clo1313_1768	Clo1313_1983
Clo1313_1604		Clo1313_0851		Clo1313_1990
Clo1313_2189		Clo1313_0849		Clo1313_1971
Clo1313_1955		Clo1313_2234		Clo1313_0685
Clo1313_1960		Clo1313_2530		Clo1313_0501
Clo1313_0350		Clo1313_2635		Clo1313_2022
Clo1313_1694		Clo1313_2795		Clo1313_2479
Clo1313_1396		Clo1313_2856		Clo1313_2188
Clo1313_1816		Clo1313_0177		Clo1313_1494
Clo1313_1425		Clo1313_0521		Clo1313_0693
Clo1313_1786		Clo1313_0563		Clo1313_2858
		Clo1313_1564		Clo1313_2859
		Clo1313_2793		
		Clo1313_2794		
		Clo1313_2860		
		Clo1313_2861		
		Clo1313_0399		
		Clo1313_0522		
		Clo1313_2857		
