# Supplementary material for: How does cellulosome composition influence deconstruction of lignocellulosic substrates in Clostridium (Ruminiclostridium) thermocellum DSM 1313?
Source: Biotechnol Biofuels. 2017 Sep 18;10:222. doi: 10.1186/s13068-017-0909-7 (PMC5604425; doi:10.1186/s13068-017-0909-7)
Supplement: Supplementary file 4 — Additional file 4: Table S2. Activities of C. thermocellum cellulosomal enzymes. Recombinant C. themocellum cellulosomal (type I dockerin-containing) enzymes were a kind gift of CelDezyner Ltd. (Rehovot, Israel). Activity assays were conducted in a final volume of 1 ml, containing 50 mM acetate buffer, 1% carboxymethyl cellulose (sodium salt, low viscosity CMC, BDH chemicals) and 7 nM enzyme. Samples were incubated with shaking for 3 h at 60 °C. Released soluble sugar (reducing ends) concentrations were analyzed by the dinitrosalicylic acid (DNS) method, as previously described [36]. Final soluble sugar concentrations were determined against a glucose calibration curve, and CMCase activities [µM reducing ends·µmol enzyme−1·min−1] were calculated. *A thermostable clone of Cel8A [66] was used. [file 13068_2017_909_MOESM4_ESM.pdf]

Additional file 4

Table S2. **Activities of *C. thermocellum* cellulosomal enzymes**. Recombinant *C. themocellum* cellulosomal (type I dockerin-containing) enzymes were a kind gift of CelDezyner Ltd (Rehovot, Israel). Activity assays were conducted in a final volume of 1 ml, containing 50 mM acetate buffer, 1% carboxymethyl cellulose (sodium salt, low viscosity CMC, BDH chemicals) and 7 nM enzyme. Samples were incubated with shaking for 3 h at 60°C. Released soluble sugar (reducing ends) concentrations were analyzed by the dinitrosalicylic acid (DNS) method, as previously described [1]. Final soluble sugar concentrations were determined against a glucose calibration curve, and CMCase activities [μM reducing ends·μmol enzyme^{-1}·min^{-1}] were calculated. * A thermostable clone of Cel8A [2] was used.

Enzyme		CMCase activity [μmol reducing ends·(μmol enzyme)$^{-1}$·min^{-1}]
Clo1313_1604	Cel9/44J	3016.03
Clo1313_1396	Cel9D	2848.15
Clo1313_1960	Cel8A*	1635.19
Clo1313_1694	Cel9F	666.18
Clo1313_2189	Cel9N	654.21
Clo1313_1816	Cel5L	577.39
Clo1313_1425	Cel5E	574.06
Clo1313_0413	Cel5G	499.15
Clo1313_2805	Cel5O	450.38
Clo1313_0851	Xgh74A	448.92
Clo1313_1659	Cel9R	136.17
Clo1313_0177		110.90
Clo1313_3023	Cel9U	76.01
Clo1313_0521	Xyn11A	70.60
Clo1313_1955	Cel9P	59.55
Clo1313_0399		53.06
Clo1313_2530	Xyn10C	37.05
Clo1313_1398		29.66
Clo1313_2794		20.04
Clo1313_0987	Xyn43D	6.83
Clo1313_2860		4.91
Clo1313_1808	Cbh9A	4.88
Clo1313_0501		2.82
Clo1313_1809	Cel9K	0.85
Clo1313_2858		0.00

References

1. Miller GL. Use of dinitrosalicylic acid reagent for determination of reducing sugar. Anal Biochem. 1959;31:426–428.

2. Anbar M, Gul O, Lamed R, Sezerman UO, Bayer EA. Improved thermostability of *Clostridium thermocellum* endoglucanase Cel8A by using consensus-guided mutagenesis. Appl Environ Microbiol. 2012;78:3458–3464.
