# Supplementary material for: How does cellulosome composition influence deconstruction of lignocellulosic substrates in Clostridium (Ruminiclostridium) thermocellum DSM 1313?
Source: Biotechnol Biofuels. 2017 Sep 18;10:222. doi: 10.1186/s13068-017-0909-7 (PMC5604425; doi:10.1186/s13068-017-0909-7)
Supplement: Supplementary file 5 — Additional file 5: Table S3. Soluble (non-cellulosomal) carbohydrate-active enzyme composition. Soluble (dockerin-lacking) carbohydrate-active enzyme compositions of the different cellulosomes were analyzed by label-free LC–MS/MS mass spectrometry. The intensities were normalized by the intensity-based absolute quantification (iBAQ) method. The resultant iBAQ intensities were divided by the iBAQ intensity of ScaA in each sample, thereby generating a relative abundance index. Standard deviations of duplicate samples of CB- and MCC-derived cellulosomes and triplicates of glucose-, alSG-, alCS-, and acCS-derived cellulosomes were analyzed. Gene ID and CAZy annotation of the subunits are designated. Acronyms: GH, glycoside hydrolase; CBM, carbohydrate-binding module; CE, carbohydrate esterase; GT, glycosyl transferase. [file 13068_2017_909_MOESM5_ESM.pdf]

Additional file 5

Table S3. Soluble (non-cellulosomal) carbohydrate-active enzyme composition. Soluble (dockerin-lacking) carbohydrate-active enzyme compositions of the different cellulosomes were analyzed by label-free LC-MS/MS mass spectrometry. The intensities were normalized by the intensity-based absolute quantification (iBAQ) method. The resultant iBAQ intensities were divided by the iBAQ intensity of ScaA in each sample, thereby generating a relative abundance index. Standard deviations of duplicate samples of CB- and MCC-derived cellulosomes and triplicates of glucose-, alSG-, alCS-, and acCS-derived cellulosomes were analyzed. Gene ID and CAZy annotation of the subunits are designated. Acronyms: GH, glycoside hydrolase; CBM, carbohydrate-binding module; CE, carbohydrate esterase; GT, glycosyl transferase.

		Glucose		CB		MCC		alSG		alCS		acCS	
Clo1313_0054	CBM3	9.44E-03	± 0.005038	1.07E-01	± 0.031462	1.31E-01	± 0.01	1.08E-01	± 0.024918	6.23E-02	± 2.72E-02	1.96E-01	± 0.033233
Clo1313_0394	CBM16,CBM16	2.10E-03	± 0.002362	2.27E-02	± 0.007412	1.15E-01	± 0.03	9.08E-03	± 0.003848	2.30E-03	± 1.89E-03	2.93E-03	± 0.001209
Clo1313_0647	CBM16	4.16E-01	± 0.119481	7.35E-01	± 0.296293	6.29E-02	± 0.02	2.71E-02	± 0.015787	1.39E-01	± 4.46E-02	8.48E-03	± 0.001953
Clo1313_2584	CBM6	1.64E-03	± 0.000966	4.29E-02	± 0.007443	1.81E-02	± 0.01	2.01E-02	± 0.005801	6.09E-03	± 3.88E-03	3.07E-02	± 0.016519
Clo1313_1139	CE4	1.90E-03	± 0.002241	5.29E-03	± 0.005859	1.29E-02	± 0.01	1.57E-03	± 0.001584	3.10E-04	± 2.89E-04	7.58E-04	± 0.000893
Clo1313_2473	CBM50,CBM50,GH18	0.00	± 0.00	4.21E-04	± 0.000595	1.15E-02	± 0.00	6.52E-05	± 0.000113	1.85E-04	± 3.20E-04	2.49E-04	± 0.000346
Clo1313_1927	CBM50	0.00	± 0.00	0.00	± 0.00	9.91E-03	± 0.00	0.00	± 0.00	0.00	± 0.00	0.00	± 0.00
Clo1313_0397	CBM54,GH16,CBM4,CBM4,CBM4,CBM4,CBM4	9.81E-02	± 0.010329	1.10E-01	± 0.018171	9.19E-03	± 0.00	8.57E-03	± 0.002492	1.66E-02	± 4.29E-03	8.16E-03	± 0.003153
Clo1313_1958	CBM3	5.77E-04	± 0.000746	3.95E-03	± 0.002357	4.93E-03	± 0.00	1.21E-04	± 6.74E-05	0.00	± 0.00E+00	4.00E-04	± 0.000274
Clo1313_2433	GT51	5.55E-03	± 0.002327	1.30E-02	± 0.006655	4.62E-03	± 0.00	2.26E-03	± 0.001111	2.30E-03	± 9.66E-04	5.10E-03	± 0.001969
Clo1313_1867	GT35	7.84E-05	± 0.000136	6.58E-04	± 1.73E-05	4.61E-03	± 0.00	3.14E-04	± 4.23E-05	1.79E-04	± 1.48E-04	4.77E-05	± 4.23E-05
Clo1313_0436	GH18	5.04E-03	± 0.002955	2.88E-02	± 0.008813	4.27E-03	± 0.00	3.02E-03	± 0.000797	3.31E-03	± 1.46E-03	6.00E-03	± 0.001255
Clo1313_2777	GH10	3.73E-04	± 0.000253	1.37E-02	± 0.003649	2.83E-03	± 0.00	1.65E-03	± 0.000682	1.17E-03	± 6.49E-04	2.20E-03	± 0.001489
Clo1313_1128	GT4	0.00	± 0.00	0.00	± 0.00	2.65E-03	± 0.00	2.42E-05	± 4.19E-05	0.00	± 0.00E+00	0.00	± 0.00
Clo1313_2173	CBM3	1.96E-04	± 0.000209	6.50E-03	± 0.002443	1.34E-03	± 0.00	2.17E-04	± 7.33E-05	2.91E-04	± 1.83E-04	1.70E-04	± 0.000193
Clo1313_0646	CBM16,GT39	1.12E-03	± 0.001246	4.63E-03	± 0.002995	1.01E-03	± 0.00	4.65E-04	± 0.000225	9.02E-04	± 2.97E-04	4.70E-04	± 0.00021
Clo1313_2999	GT4	1.14E-03	± 0.001369	9.42E-03	± 0.012261	8.46E-04	± 0.00	1.42E-03	± 0.00242	1.69E-03	± 2.24E-03	2.09E-04	± 0.000198
Clo1313_1947	GT5	2.67E-04	± 0.000462	1.00E-04	± 0.000142	6.52E-04	± 0.00	3.10E-05	± 5.37E-05	1.15E-05	± 1.99E-05	0.00	± 2.33E-05
Clo1313_2854	CBM48,GH13	2.63E-05	± 4.56E-05	0.00	± 0	5.32E-04	± 0.00	0.00	± 0	0.00	± 0.00E+00	0.00	± 0.00
Clo1313_0090	CBM3	6.21E-04	± 0.000266	7.28E-03	± 0.003453	4.26E-04	± 0.00	1.36E-03	± 0.000389	4.09E-03	± 1.05E-03	1.96E-04	± 9.12E-05
Clo1313_0645	GT2	1.91E-02	± 0.031255	1.17E-02	± 0.016505	3.32E-04	± 0.00	0.00	± 0	1.46E-02	± 2.00E-02	0.00	± 0.007352
Clo1313_2020	GH1	4.26E-04	± 0.000557	6.41E-03	± 0.000207	2.42E-04	± 0.00	5.53E-05	± 4.8E-05	0.00	± 0.00E+00	0.00	± 0.00
Clo1313_1962	CBM3	0.00	± 0.00	0.00	± 0.00	9.08E-05	± 0.00	9.48E-05	± 0.000164	2.20E-05	± 3.81E-05	8.70E-04	± 0.000235
Clo1313_0539	GH94	1.75E-04	± 0.000162	1.96E-03	± 0.002106	5.26E-05	± 0.00	1.01E-05	± 1.76E-05	1.44E-05	± 1.35E-05	5.33E-06	± 9.23E-06
Clo1313_1001	CBM3,CBM4	4.17E-04	± 0.000433	3.03E-03	± 0.001113	0.00	± 0.00	2.75E-03	± 0.00197	5.55E-03	± 2.24E-03	1.05E-03	± 0.000647
Clo1313_0333	GH23	3.69E-04	± 0.00032	6.34E-04	± 0.000896	0.00	± 0.00	1.09E-04	± 0.000189	2.09E-04	± 3.61E-04	4.78E-04	± 0.000406
Clo1313_0485	GH18	8.18E-05	± 8.88E-05	1.22E-02	± 0.013901	0.00	± 0.00	1.23E-04	± 0.000114	3.59E-05	± 4.57E-05	4.32E-04	± 0.000126
Clo1313_0974	GT5	0.00	± 0.00	5.12E-05	± 7.24E-05	0.00	± 0.00	1.95E-05	± 3.38E-05	0.00	± 0.00E+00	0.00	± 0.00
